# Machine learning in the detection and management of atrial fibrillation

**DOI:** 10.1007/s00392-022-02012-3

**Published:** 2022-03-30

**Authors:** Felix K. Wegner, Lucas Plagwitz, Florian Doldi, Christian Ellermann, Kevin Willy, Julian Wolfes, Sarah Sandmann, Julian Varghese, Lars Eckardt

**Affiliations:** 1 Klinik für Kardiologie II - Rhythmologie, Universitätsklinikum Münster, Albert-Schweitzer-Campus 1, 48149 Münster, Germany; 2 Institut für Medizinische Informatik, Westfälische-Wilhelms-Universität Münster, Albert-Schweitzer-Campus 1, 48149 Münster, Germany

**Keywords:** Deep learning, Neural network, Electrophysiology, Arrhythmia, Artificial intelligence

## Abstract

Machine learning has immense novel but also disruptive potential for medicine. Numerous applications have already been suggested and evaluated concerning cardiovascular diseases. One important aspect is the detection and management of potentially thrombogenic arrhythmias such as atrial fibrillation. While atrial fibrillation is the most common arrhythmia with a lifetime risk of one in three persons and an increased risk of thromboembolic complications such as stroke, many atrial fibrillation episodes are asymptomatic and a first diagnosis is oftentimes only reached after an embolic event. Therefore, screening for atrial fibrillation represents an important part of clinical practice. Novel technologies such as machine learning have the potential to substantially improve patient care and clinical outcomes. Additionally, machine learning applications may aid cardiologists in the management of patients with already diagnosed atrial fibrillation, for example, by identifying patients at a high risk of recurrence after catheter ablation. We summarize the current state of evidence concerning machine learning and, in particular, artificial neural networks in the detection and management of atrial fibrillation and describe possible future areas of development as well as pitfalls.

**Graphical abstract:**

Typical data flow in machine learning applications for atrial fibrillation detection.

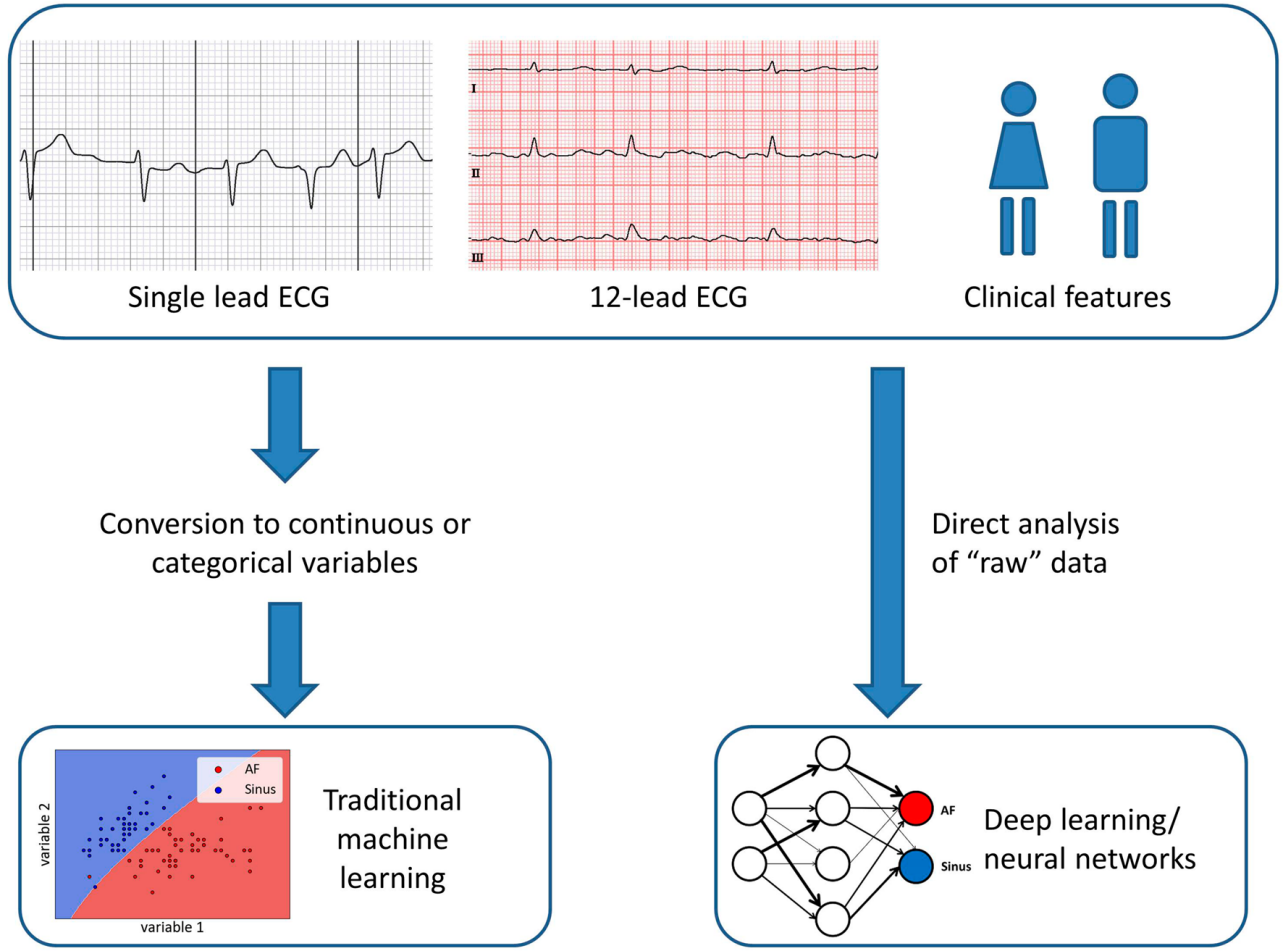

## Introduction

Machine learning (ML) constitutes a subdiscipline of artificial intelligence (AI) and is characterized by the automated detection of patterns from data. Its use is particularly advantageous in the detection of non-linear associations and modern computational power allows for the analysis of large datasets. The use of artificial neural networks has already led to transformation of scientific studies and understanding of vastly different areas such as language processing, object recognition, and predictive analysis. One important aspect is the increasing availability of large standardized digitized datasets, for example, electronic health records or imaging and ECG databases.

Atrial fibrillation (AF) is the most common sustained arrhythmia in adults and characterized by unorganized electrical activation leading to ineffective mechanical contraction of the atria [1]. While a substantial number of patients describe symptoms such as palpitations or tachycardia, many episodes remain asymptomatic and therefore only lead to a medical diagnosis if complications arise. An especially severe complication is the occurrence of thromboembolic events and stroke, which results in significant morbidity and mortality worldwide [2]. This risk can be ameliorated if a diagnosis is reached early and anticoagulation is established in patients with risk factors for thromboembolic events. Therefore, medical associations have stressed the importance of AF screening and current European guidelines recommend opportunistic screening of all adults > 65 years of age [1]. Nonetheless, many patients are diagnosed with AF only after a thromboembolic event has occurred [3, 4]. Novel technologies such as machine learning may aid clinicians in identifying patients at a high risk of AF and may consequently improve patient outcome by reducing thromboembolic complications [5]. The aim of this review is to summarize currently available data and areas of potential future development as well as risks and pitfalls in the integration of machine learning into AF management.

## Overview of machine learning methods

**Fig. 1:**
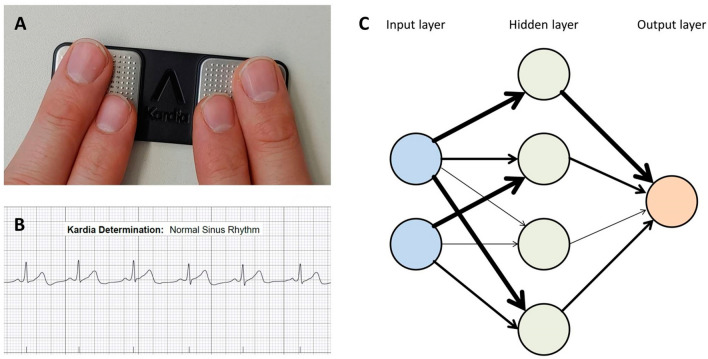
Panels A and B: Illustration of a smartphone-based ECG device (**A**) with an automated rhythm classification based on traditional machine-learning algorithms (**B**). Panel C: schematic depiction of a simple neural network designed with one hidden layer. The width of connecting arrows signifies differently weighted connections between layers

Artificial intelligence describes the ability of technical constructs such as computers to independently process data and reach conclusions which typically require human cognitive function [6]. To this end, input data need to be machine-readable, most preferably in a highly structured form. Table 1 lists a selection of different machine learning methods and their corresponding clinical applications in AF detection and management. Traditional methods of AI such as supervised machine learning have been used for decades and include algorithms such as random forests or support vector machines. For this type of model, a tabular data structure is required to detect patterns in parameters such as age, corrected QT time, or heart rate variability. Supervised machine learning can be utilized to detect both linear and non-linear relationships within data, but is dependent on a human operator labeling data and selecting input variables. According to the form of the labels, continuous values lead to a regression analysis, in contrast to fixed classes of a classification problem. Conversely, unsupervised learning refers to analyses that detect clusters based on similarities in the absence of labeled data. After successful training of machine learning models, considerably less computational power is needed for their execution. Hence, previously trained and tested machine learning methods can be integrated into wearable technology and smartphone applications (see Fig. 1, panels A and B).

**Table 1:** Machine learning methods and corresponding applications in the detection and management of atrial fibrillation

Machine learning method	Description	Example application	Reference
Traditional machine learning			
Cox regression	Probability distribution estimating time to a pre-specified event	Prediction of post-ablation AF recurrence	[55]
Support vector machine	Utilizes hyperplane to separate two classes non-linearly	AF detection through HRV analysis of photoplethysmography readings	[23]
Random forest	Average of hierarchical decision trees’ interpretation	Locating re-entrant drivers in AF	[56]
Deep learning			
Convolutional neural network	Mimics biological neural networks by incorporating nodes processing data in a hierarchical fashion	Detection of AF from a sinus-rhythm 12-lead ECG	[32]

*AF* atrial fibrillation, *HRV* heart rate variability

As a powerful technology, deep learning utilizes artificial neural networks to independently identify features in the input data and therefore to detect previously unknown patterns too. Artificial neural networks are computational structures modeled after biological neural systems such as the human brain [7]. In its most basic form, an artificial neural network connects input data directly to an output layer. More complex systems such as deep neural networks (DNN) contain multiple layers, which may perform different tasks and whose strength of connection is determined by trainable and adjustable weights, while not all nodes of neighboring layers may be connected (see Fig. 1, panel C) [8]. Depending on the input data, adapted network architectures indicate great predictive potential, e.g. convolutional neural networks for images, ECG or time series. This special type of network is characterized by convolutional layers using filters to identify data features such as edges or curves in images, which are combined into feature maps [9].

In supervised machine learning, data are commonly split into separate training and test sets. The learning algorithm is first trained using the training dataset including varied data with appropriate labels for its planned purpose. In addition to both sets, training of neural networks is usually monitored by a third set, the validation set. This is used to detect shortcomings of the algorithm before the final evaluation of the neural network is conducted by assessing its predictive accuracy in an analysis of the test set. Further literature details computational foundations outside the scope of this review as well as limitations in the implementation of individual machine learning methods [10–13].

## Clinical applications

In recent years, medical research has been transformed using machine learning approaches. The applications can be divided into strategies for the detection of AF and approaches improving the care of patients with known AF.

### Screening for atrial fibrillation

Atrial fibrillation detection plays an important role in the avoidance of mortality and morbidity associated with AF. To this end, traditional machine learning methods have been used in clinical practice for decades and include, for example, automated ECG machine diagnoses. Novel approaches have included transferring this well-known technology to mobile devices such as smartphones and wearable technology. While smartphone-based ECG devices have been developed by multiple companies [14], the AliveCor Kardia AF detection algorithm has been evaluated most frequently by scientific studies and was shown to have a high negative predictive value for the presence of AF [15–22]. Alternatively, smartphone applications such as the Cardiio app utilize photoplethysmographic measurements obtained through a built-in smartphone camera and was shown to be both sensitive and specific for the detection of AF in a recent publication by Yan et al. [23]. However, currently, only two large-scale prospective studies exist on the utility of smart-technology based AF screening [24, 25]. The Apple Heart study was conducted as a prospective, siteless study including 419,000 participants with an Apple smartwatch residing in the United States. Study subjects monitored their heart rhythm with a photoplethysmographic sensor and if a recording was interpreted by the automated algorithm as probable AF, a 7 day ECG screening was conducted by a mailed ECG patch [24]. Of the 2161 participants (0.52%) who received a notification of irregular heart rhythm, 450 participants (21%) returned their ECG patches for analysis and AF was present in 34% of returned recordings. Similarly, the Huawei heart study included almost 190,000 Chinese participants who monitored their heart rhythm with a Huawei smartwatch-based photoplethysmographic algorithm [25]. While 424 subjects (0.23%) received an automated algorithm interpretation of suspected AF, 262 (62%) were effectively followed up by either 12-lead or Holter ECG. Of those, 227 (87%) were confirmed to have a diagnosis of AF. These studies show the potential, but also the limitations of large population-based smart-technology screening programs, with an acceptable positive predictive value but also a significant loss of participants to clinical follow-up. Additionally, because of the study designs, the rate of false-negatives (i.e., persons with unknown AF and no photoplethysmographic recordings suspected to be AF) could not be reported.

Various studies have evaluated the utility of deep neural networks in AF screening. At least two working groups independently evaluated a DNN in the interpretation of smartwatch plethysmographic data und were able to document a significantly increased sensitivity and specificity compared to previously presented studies utilizing traditional machine learning methodology [26, 27]. Concerning diagnosis from a one-lead ECG recording, Hannun et al. showed a superior accuracy of a DNN compared with board-certified cardiologists [28], which furthermore extended to other arrhythmias such as regular supraventricular tachycardia and atrioventricular block. Unifying applications across different diagnostic modalities, Ramesh et al. recently reported the development of a DNN able to detect AF with a high diagnostic accuracy in both ECG and photoplethysmographic recordings [29]. Importantly, neural networks can also aid the screening for AF even when it is absent at the time of presentation. For example, different groups were able to improve previous classical risk stratification models by utilizing DNNs to estimate the likelihood of AF occurrence in high-risk populations such as patients with chronic kidney disease [30] or patients with a history of ischemic stroke [31]. In a landmark publication, Attia et al. recently described a DNN trained on almost 650,000 ECG of 180,000 patients capable of detecting patients with AF from a sinus rhythm ECG [32]. The DNN was trained on ECG obtained at the Mayo Clinic Rochester between 1993 and 2017 and sinus rhythm recordings were deemed to show a patient with AF if it was first documented within 31 days of the sinus rhythm recording. The authors were able to show a sensitivity of 79% and a specificity of 79.5% of the DNN in the detection of AF-patients from the sinus rhythm ECG, which further increased to 82.3% and 83.4%, respectively, when multiple ECG from the same patient were analyzed. A similar recent publication was able to considerably increase the diagnostic window by evaluating a DNN capable of detecting AF onset within one year of the recording of an index sinus rhythm ECG [33].

### Management of patients with atrial fibrillation

Concerning the management of patients with known AF, multiple studies have evaluated the utility of both traditional machine learning algorithms and neural networks for the improvement of patient care. Handheld cardiac devices were shown to have a comparable diagnostic accuracy to traditional Holter monitors in the detection of AF recurrences after catheter ablation [34] and a deep neural network was able to estimate the plasma concentration of a class III antiarrhythmic drug from the analysis of a 12-lead ECG [35]. Various working groups developed neural networks with accuracy higher than previous risk scores and traditional linear or logistic algorithms in the risk assessment of AF recurrence after either catheter ablation [36, 37] or thoracoscopic ablation for AF [38], possibly allowing for the identification of patients with a higher need for close follow-up. Concerning procedural aspects of catheter ablation, two separate working groups were able to train and evaluate DNNs for identification of nonpulmonary AF triggers from intracardiac electrograms obtained during an ablation procedure [39, 40], which may be targeted by ablation in AF cases refractory to pulmonary vein isolation. Interestingly, Li et al. recently published a study evaluating an algorithm developed to detect AF episodes associated with rapid ventricular rate and low physical activity, which may include the most symptomatic AF episodes [41]. The authors were able to show that the developed algorithm was able to detect AF episodes up to 4.5 min before onset, possibly allowing for the development of algorithm-guided interventions before the onset of an AF episode, such as the intake of antiarrhythmic medication. Additionally, DNNs were shown to improve the estimation of AF-associated risks with working groups being able to show an improvement of estimation of all-cause mortality [42] and neurological outcome after an AF-related stroke [43]. A recent DNN-based analysis of pooled data from nine double-blinded, randomized, placebo-controlled trials evaluating betablockers in heart failure was also able to detect a mortality benefit in young patients with reduced LVEF and AF [44].

## Pitfalls and risks in the application of machine learning

Mobile health devices and wearable technology including traditional machine learning algorithms are currently being integrated into clinical practice across different health systems throughout the world. In contrast, multiple issues impede the widespread implementation of deep neural networks into routine clinical care.

Since most algorithms developed for the detection of AF rely on the recognition of absolutely irregular R-R intervals, these have a high likelihood of missing cases of atrial flutter. In contrast to AF, atrial flutter is commonly symptomatic because of a rapid ventricular response and is therefore more likely diagnosed through conventional clinical pathways. Nonetheless, both arrhythmias confer a similar risk of thromboembolism and their possible underdiagnosis by automated algorithms may undermine confidence in their reliability. To this end, a recent publication was able to train a DNN to correctly identify both AF and cavotricuspid isthmus-dependent atrial flutter [45].

Additionally, major concerns persist about the intransparency of DNN models in clinical practice, which may provide physicians with highly relevant information but little to no explanation about how a conclusion was reached. Different working groups have attempted to ameliorate this problem. For example, Tison et al. combined different machine learning methods to create a personalized ECG vector profile able to estimate values such as LV mass and e’ velocity while simultaneously annotating the ECG sections most important for the individual value, therefore enabling clinicians to check the results for plausibility [46]. Similarly, Mousavi et al. recently described a DNN able to distinguish AF from sinus rhythm while simultaneously highlighting the most relevant areas of the respective ECG for rhythm discrimination [47]. However, easily interpretable DNN models may offer only a reduced clinical benefit when directly compared to more complex models which lack transparency [48].

Concerning algorithm-based screening for AF, doubts persist about the transferal of previous findings of thromboembolic risk onto these patients with often short-lasting, asymptomatic episodes. Importantly, a recent large study evaluating loop-recorder based AF screening in patients with risk factors for stroke was unable to find an improvement in the prevention of thromboembolic events even though patients with a loop-recorder were three times as likely as a control group to be diagnosed with AF [49]. Similarly, patients who are found to have AF through photoplethysmographic continuous rhythm monitoring or during screening advised by an automated DNN-based risk assessment may conceivably have a lower thromboembolic risk than patients diagnosed with AF through a traditional clinical pathway and indications for anticoagulation may have to be re-evaluated.

## Future developments

While diverse applications for machine learning and artificial neural networks have been described, no large prospective studies have evaluated an impact of these technologies on hard clinical endpoints such as thromboembolic events or mortality. Although the question of direct clinical impact, especially concerning anticoagulation in newly diagnosed AF patients, should warrant further investigation, no prospective study on this subject has been registered at clinicaltrials.gov as of January 4, 2022. However, both artificial neural networks and mobile health applications have the potential to change clinical practice in the near future and a recent position paper by EHRA and ESC working groups was the first to specifically comment on these novel technologies [50]. One further application specific to DNNs may be the optimization of health-system wide workflows and generalized risk assessment at the primary care level [51].

A perspective on the future of artificial intelligence in the diagnosis and treatment of atrial fibrillation may be obtained by looking at recent advances in cardiac imaging. While echocardiography and magnetic resonance images have traditionally been manually acquired and interpreted, studies have recently been able to show the feasibility of AI-guided image acquisition [52] and automated interpretation [53] with a quality at least equal to that of human investigators and much improved speed [54]. In light of these advances, completely automated image acquisition and especially interpretation with a physician only supervising and validating results seems likely.

## Conclusion

Machine learning has the potential to transform medical practice in general and the screening for and management of patients with AF in particular in the near future. In this regard, novel technologies such as mobile, automated screening for AF and the utilization of artificial neural networks allow for approaches to AF care not possible with traditional technologies. Obstacles in the application of machine learning into routine clinical practice which should be addressed by future studies include the intransparency of neural networks and the lack of evidence showing an improvement in clinical outcomes.
